# Electrospun PCL Patches with Controlled Fiber Morphology and Mechanical Performance for Skin Moisturization via Long-Term Release of Hemp Oil for Atopic Dermatitis

**DOI:** 10.3390/membranes11010026

**Published:** 2020-12-31

**Authors:** Sara Metwally, Daniel P. Ura, Zuzanna J. Krysiak, Łukasz Kaniuk, Piotr K. Szewczyk, Urszula Stachewicz

**Affiliations:** Faculty of Metals Engineering and Industrial Computer Science, AGH University of Science and Technology, 30-059 Krakow, Poland; metwally@agh.edu.pl (S.M.); urad@agh.edu.pl (D.P.U.); krysiak@agh.edu.pl (Z.J.K.); kaniuk@agh.edu.pl (Ł.K.); pszew@agh.edu.pl (P.K.S.)

**Keywords:** PCL, electrospinning, fibers, tensile strength, hemp oil, skin patches, release, skin moisture, atopic dermatitis

## Abstract

Atopic dermatitis (AD) is a chronic, inflammatory skin condition, caused by wide genetic, environmental, or immunologic factors. AD is very common in children but can occur at any age. The lack of long-term treatments forces the development of new strategies for skin regeneration. Polycaprolactone (PCL) is a well-developed, tissue-compatible biomaterial showing also good mechanical properties. In our study, we designed the electrospun PCL patches with controlled architecture and topography for long-term release in time. Hemp oil shows anti-inflammatory and antibacterial properties, increasing also the skin moisture without clogging the pores. It can be used as an alternative cure for patients that do not respond to traditional treatments. In the study, we tested the mechanical properties of PCL fibers, and the hemp oil spreading together with the release in time measured on skin model and human skin. The PCL membranes are suitable material as patches or bandages, characterized by good mechanical properties and high permeability. Importantly, PCL patches showed release of hemp oil up to 55% within 6 h, increasing also the skin moisture up to 25%. Our results confirmed that electrospun PCL patches are great material as oil carriers indicating a high potential to be used as skin patches for AD skin treatment.

## 1. Introduction

Atopic dermatitis (AD) is a chronic, inflammatory skin disease affecting numbers of children and adults, with worldwide prevalence ranging from 0.2%–24.6% [1]. AD is characterized by itchy, inflamed, dry skin, and is caused by various irritants and allergens [2,3]. The pharmaceutical compounds (drugs) have to be delivered to the right area, at the right time and concentration to accomplish the desired therapeutic effect [4]. In current AD treatments, there is a lack of long-term cures, therefore the development of new strategies for skin regeneration is needed. Different drug delivery systems including nanoparticles, cryogel-based formulations, microneedle patches, and nanoemulsions were developed in pharmaceutical and cosmetic industries to treat it [5]. Also, skin patches gain a lot of interest in cosmetic, topical, and transdermal delivery systems, being classified into the reservoir systems and the drug dissolved or dispersed in an adhesive layer. In the first one, the drug is dissolved or dispersed in a reservoir, where the release rate is controlled by a membrane or matrix. In the second sub-type, the drug is dissolved or dispersed in an adhesive layer which is in contact with the skin [6]. Fibrous membranes, scaffolds, and patches have been widely utilized in tissue engineering [7,8,9], hemostats [10], tendon repair [11], drug delivery systems for wound healing [12,13], and in the face masks applications [14,15]. Importantly, the scaffold must show adequate mechanical properties, to provide robust support to the damaged tissue. Among the wide range of polymers, PCL has been extensively used in skin bioengineering due to its biocompatibility, desirable mechanical, and biodegradable properties [16,17]. Additionally, it is a soft- and hard tissue biocompatible material, and blended with collagen is suitable for the attachment and proliferation of human dermal fibroblasts, showing great potential for the treatment of skin tissue defects and burn injuries [18]. PCL fibers were also designed as a support for cosmetic patches, containing alginate and Spirulina, typical marine resources used in cosmetic products. The Spi/Alg-PCL composite showed no cytotoxicity for human keratinocyte cells and expressed more moisture and better adhesiveness compared to alginate patches [19].

Often, people suffering from AD, do not respond to traditional therapies, and cannot tolerate even basic soothing or moisturizing formulas. Therefore, an alternative cure needs to be developed to treat the AD flare-ups. Natural oils have been shown to have both anti-inflammatory, antimicrobial, and barrier restoring effects [20]. Hempseed has been widely utilized for thousands of years in the treatment of various disorders in traditional, oriental medicine. Hempseed oil contains over 80% polyunsaturated fatty acids (PUFAs) and is extremely rich in essential fatty acids (EFAs) linoleic acid (18:2 omega-6) and alpha-linolenic acid (18:3 omega-3). The two main proteins in hempseed are edestin and albumin [21]. It was shown that the ingestion of hempseed oil increases the EFAs, linoleic acids, and improves the dryness and itchiness of atopic skin [22]. Previous clinical studies demonstrated that topically applied hemp oil is beneficial in mucosal skin wound healing [23]. Hempseed oil reactivates the natural barrier function to protect the dry and scaly skin while boosting the moisture balance.

The study aimed to design PCL fiber membranes with controlled hemp oil release and appropriate mechanical properties for skin patch applications. In our study, we performed the electrospinning of four types of PCL patches based on smooth, porous, random, and aligned fibers. We examined the mechanical properties of PCL patches together with their oil wetting properties. Our results demonstrated that PCL fibers show high tensile strength and strain properties. Additionally, the porous fibers formed the interlocking systems that increased their mechanical properties, as it was shown with fiber testing in situ in SEM. We tested the hempseed oil spreading and release from PCL patches deposited onto the skin model and human skin. We showed that oil release and spreading strongly depend on fiber morphology and orientation affecting skin moisturization. The designed PCL constructs exhibit a controlled release in a long period making them suitable for atopic skin treatment patches.

## 2. Materials and Methods

### 2.1. Materials and Electrospinning

For smooth fibers production poly (ε-caprolactone) (PCL) (CAPA 6500, M_w_ = 50,000 g·mol^−1^, Perstorp, Lowton, UK) was dissolved in chloroform to produce 18% solution in chloroform. Porous fibers were produced from 12% PCL solution in a mixture of chloroform and dimethylsulfoxide (DMSO) (POCH, Gliwice, Poland) in a ratio 90:10 *v/v*. Solutions were stirred for 2 h with a constant speed of 600 rpm and a relative temperature of 25 °C. PCL fibers were electrospun using the electrospinning apparatus EC–DIG (IME Technologies, Waalre, The Netherlands) with the parameters presented in Table 1. The fiber deposition time was kept constant (4 h) for all types of PCL samples.

### 2.2. Scanning Electron Microscopy (SEM) and Mechanical Testing

**Figure 1:**
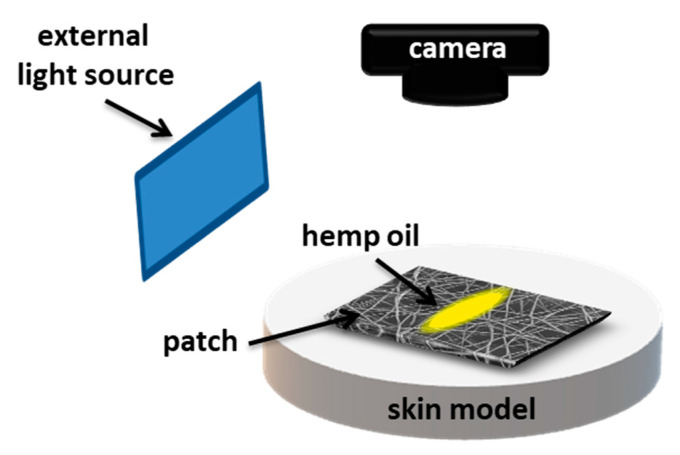
Schematic of the experimental set-up showing oil spreading on electrospun patches placed on gelatin-based skin model.

Samples were coated with approximately 5 nm Au layer using rotary-pumped sputter coating (Q150RS, Quorum Technologies, Lewes, UK). SEM (Merlin Gemini II, Zeiss, Munich, Germany) was used for imaging, applying a current of 20 pA and voltage of 3 kV. Fiber diameters (Figure 1) and sample thickness (see Figure S1 in the Supplementary Materials) were measured from SEM images using Fiji (Life-Line Version 2.0, Bethesda, MD, USA).

The mechanical properties of PCL fiber mats were measured using a tensile module with 1 N load cell (Kammrath Weiss GmbH, Dortmund, Germany). The tensile module is shown in Figure S2 in the Supplementary Material. The fiber mats were placed within the frames of a 2 mm × 1.7 mm area with cut sides. Mechanical tests were performed uniaxially with an extension speed of 50 μm·s^−1^. Maximum stress and strain were calculated from stress-strain curves using Origin Integrate Function.

### 2.3. Skin Model Preparation, Oil Spreading, and Release Tests

The skin model was prepared by dissolving gelatin (Sigma Aldrich, Haverhill, UK) in deionized water (Hydrolab, Poznań, Poland) heated up to 55 °C to obtain a 10% *w/w* solution. Next, glycerol (POCH, Gliwice, Poland) heated up to 45 °C was added to the gelatin to prepare the final 6.5/3.5 (*w/w*) solution that was stirred with a constant speed of 500 rpm for 10 min. The mixture was cast into Petri dishes and dried in the fume hood for 3 days in ambient conditions.

To examine the spreading and release of oil on PCL patches we used hemp seed oil with surface-free energy (SFE) of 31.7 ± 0.6 mN·m^−1^ and viscosity of 50.6 ± 0.2 MPa·s. The SFE and viscosity were measured, as previously reported [24]. PCL patches were placed onto the skin model, and the volume of 25 µL of hemp oil was deposited per sample, see Figure 1. Canon EOS 700D camera with EF-S 60 mm f/2.8 Macro USM zoom lens was applied to register the oil spreading in time. Images were taken with 30 min intervals for 6 h. The surface area of oil spreading was measured using FiJi (Life-Line Version 2.0, Bethesda, MD, USA) from the recorded images. Prior the oil release tests, 4 cm × 4 cm patches were placed on a volunteer’s forearm skin, and next the 25 µL of hemp oil was deposited per tested sample. The patches were weighed before deposition and after 6 h to calculate the percentage of released oil using the following equation:

(1) $$\%\;oil\;release=\;\frac{initial\;patch\;weight\;-patch\;weight\;\mathrm{after}\;6\;h\;}{initial\;oil\;weight}*100\%$$

The moisture of the skin was measured with Hydro Pen H10 (Medelink, CA) before patch application and after its removal after 6 h at the same place. PCL patches without oil deposition were used as control samples.

### 2.4. Statistical Analysis

The average fiber diameter was calculated from 100 measurements from SEM images. Five mechanical tests were carried for all PCL patches. The average thickness of the fiber mats was calculated from five independent measurements. The average spreading and release of oil was measured from three replicates for each sample. The skin moisture was measured in three independent areas with four repetitions for each measurement. The errors are based on standard deviation.

## 3. Results

### 3.1. Morphology and Sizes of PCL Fibers

**Figure 2:**
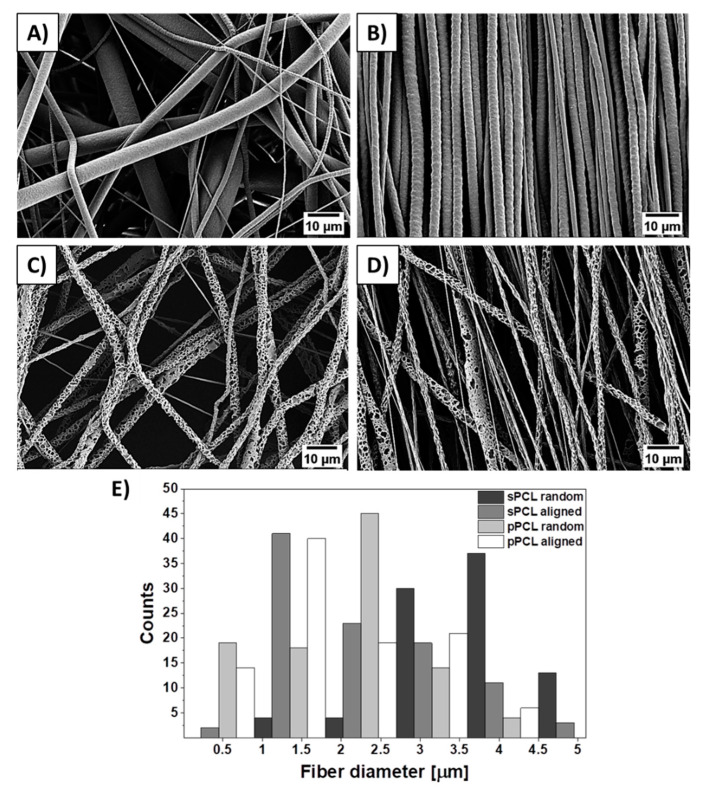
SEM micrographs of (**A**,**B**) smooth, (**C**,**D**) porous random and aligned polycaprolactone (PCL) fibers respectively, and (**E**) histogram of PCL fiber diameter distribution.

SEM images of electrospun smooth (sPCL), porous (pPCL), random, and aligned PCL fibers are shown in Figure 2. Both smooth and porous fibers show bimodal fiber diameter distribution. The average diameter of sPCL fibers were 4.3 ± 1.5 μm and 2.3 ± 1.1 μm, whereas for pPCL 1.9 ± 0.9 μm and 1.6 ± 0.8 μm for random and aligned fibers respectively. The thickness of PCL patches was measured from samples’ cross-sections obtained with freeze-fracture (see Figure S1 in the Supplementary Material). The smooth fibers shown average thickness of 196.1 ± 3.3 μm and 199.7 ± 9.2 μm, whereas porous 70.7 ± 8.7 μm and 76.4 ± 6.7 μm for random and aligned fibers respectively.

### 3.2. Mechanical Properties of PCL Fibers

**Figure 3:**
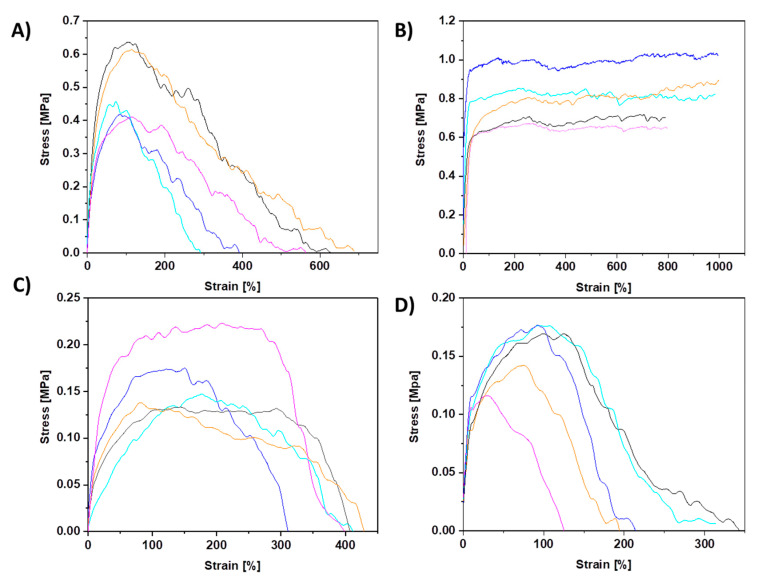
All stress–strain curves of PCL samples from tensile testing for (**A**) sPCL random, (**B**) sPCL aligned, (**C**) pPCL random, and (**D**) pPCL aligned fibers.

**Figure 4:**
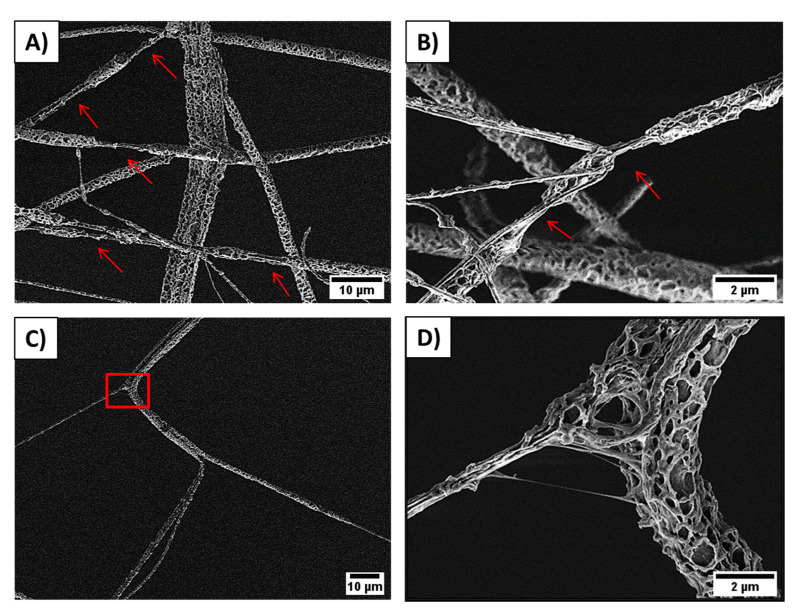
SEM images showing pPCL random fibers stretching and the formed interlocking systems between pores: (**A**) fiber necking formed after fiber interlocking, (**B**) overlapped pPCL fibers forming necking areas marked with arrows, (**C**) individual fibrils in pPCL fiber stick together and stretched, and (**D**) zoom in to the square marked in the previous image, showing the interaction between two connected fibers during the tensile testing, see also Movie S2 in the Supplementary Material.

The mechanical testing of PCL membranes revealed that sPCL fibers show a tensile strength of 0.51 ± 0.1 MPa and 0.78 ± 0.2 MPa for random and aligned fibers respectively. Aligned sPCL fiber mats have significantly higher mechanical properties i.e., strain at maximum strength and strain at failure, compared to random sPCL fibers. The strain at max stress values for pPCL fibers is similar for random and aligned samples, showing tensile strength of 0.16 ± 0.003 MPa and 0.16 ± 0.001 MPa respectively. The stress-strain curves of tensile-tested PCL fibers are shown in Figure 3, with a summary of the mechanical properties presented in Table 2, and the example of tensile tested sample under the optical microscope in the Movie S1 in the Supplementary Material. Mechanical testing of pPCL random fibers in situ in SEM revealed that fibers formed the interlocking systems (see Figure 4) due to large pores present at the surface of fibers. The interaction between the connected pPCL fibers during the tensile testing is shown in Movie S2 in the Supplementary Material.

### 3.3. Oil Spreading and Release

**Figure 5:**
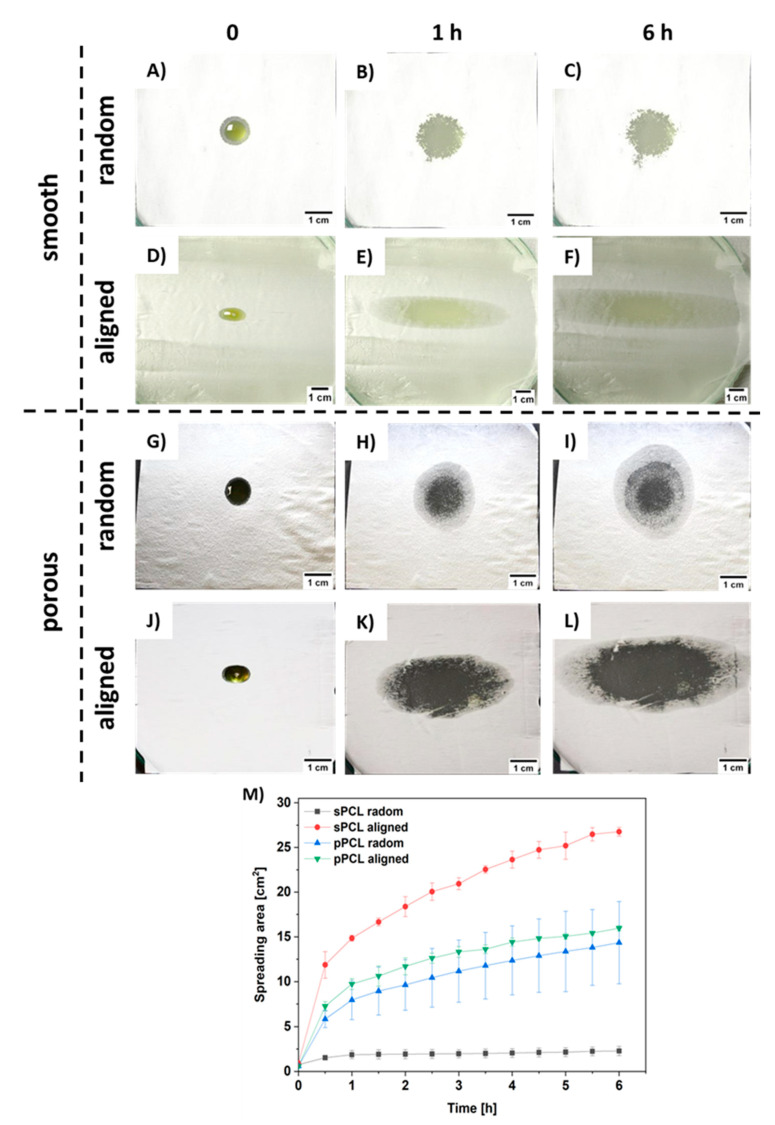
Images of hemp oil spreading s tests up to 6 h for (**A**–**C**) sPCL random, (**D**–**F**) sPCL aligned, (**G**–**I**) pPCL random, (**J**–**L**) pPCL aligned, and (**M**) graph summarizing all the spreading area of hemp oil measured every 30 min during 6 h tests.

The spreading of oil was measured on PCL patches deposited onto the gelatin-based skin model (see Figure S3 in the Supplementary Material). Within 6 h the pPCL patches showed comparable spreading area both for random and aligned fibers (Figure 5G–I,J–L). The greatest spreading area was observed for sPCL aligned fibers (see Figure 5D–F). Fiber porosity also increased the spreading area, as higher spreading was observed for pPCL compared to sPCL random fibers (Figure 5A–C,G–I). The graph showing hemp oil spreading area within 6 h is presented in Figure 5M.

**Figure 6:**
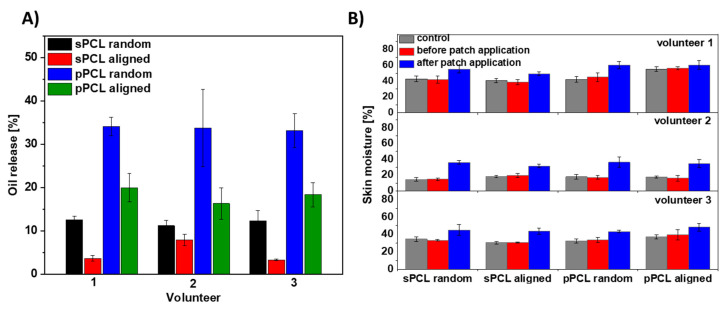
The oil test release on three volunteers’ skin (**A**) from all four types of PCL membranes: random and aligned sPCL and pPCL fibers within 6 h and (**B**) skin moisture before and after the patches’ application. The control samples are the PCL patches without the oil with the skin moisture data after the patch removal as before their application the skin moisture were at the similar levels.

**Figure 7:**
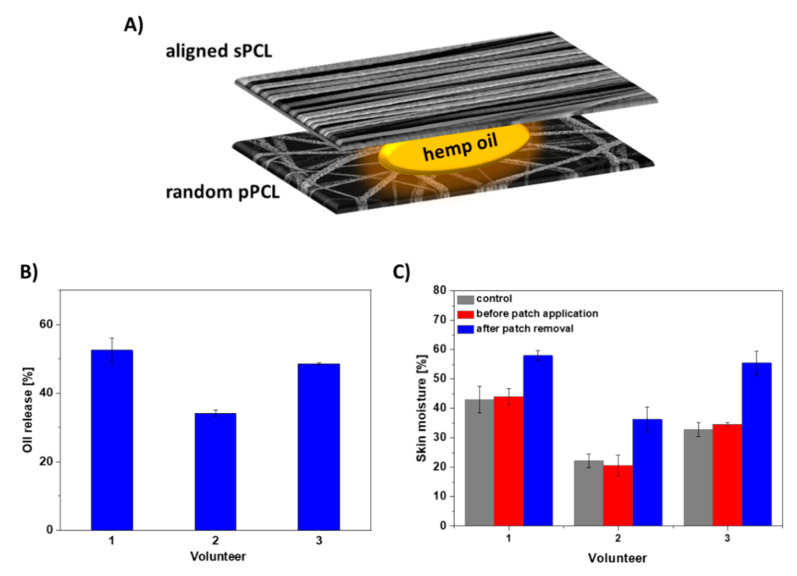
(**A**) Schematic of the layered sPCL/pPCL patches with hemp oil, (**B**) hemp oil release from layered sPCL/pPCL patches within 6 h, and (**C**) skin moisture before and after the sPCL/pPCL patch application on skin of 3 volunteers. The control sample is the layered PCL patch without the oil with the skin moisture data after the patch removal as before its application the skin moisture was at a similar level.

Additionally, the PCL patches were tested for hemp oil release on the forearm skin of three volunteers, see Figure S4 in the Supplementary Material. We measured the skin moisture before PCL patches application and after its removal after 6 h. Generally, random fibers show greater oil release in comparison to aligned samples. The higher oil release was observed for porous compared to smooth PCL fibers. The greatest release of approx. 35% was observed for pPCL random fibers for all the volunteers. The graph presenting hemp oil release from all tested PCL patches is shown in Figure 6A. Importantly, the skin moisture increased up to a maximum of 25% after the patch’s removal, see Figure 6B. The control samples were used to verify whether fiber membranes influence skin moisturization, caused by disturbance in skin thermoregulation. After removal of PCL patches without incorporation of hemp oil, skin moisture was similar to that measured before the test, therefore in Figure 6 and Figure 7 we present only the data after removing PCL patches without the oil.

We have chosen a reservoir system for designing the targeted patch based on the best mechanical properties obtained for sPCL aligned fibers together with the highest oil spreading area in time. For controlling the oil release rate in time, we selected the random pPCL fibers with the greatest oil release and higher maximum strain compared to aligned pPCL fibers. Thus, the final patch consisted of aligned sPCL fibers on the top, random pPCL fibers on the bottom with hemp oil applied between them, see the schematics of the layered sPCL/pPCL patches in Figure 7A. The designed patches increased the oil release on volunteers’ skin up to 55% within 6 h (Figure 7B). After 6 h of patch application, the volunteers’ skin showed an increase in moisture up to 20% (Figure 7C).

## 4. Discussion

The study aimed to design PCL fiber membranes differing in fiber morphology and mechanical properties to produce skin patches with controlled hemp oil release. For this purpose, we electrospun four types of PCL patches with smooth, porous, random, and aligned fibers to analyze the influence of membrane architecture on long-term oil release. Porous fibers were obtained through phase separation by the addition of DMSO to the PCL solution [25], and increasing humidity up to 70% [26]. The higher diameter of sPCL compared to pPCL fibers was mainly due to different solvents used for the preparation of polymer solution. The addition of DMSO increased polymer solution conductivity that resulted in decreased pPCL fibers diameter [25]. The 5 cm increase in distance between nozzle and collector while electrospinning of pPCL fibers, elongated the time of fibers drying and stretching leading to pPCL fiber diameter decrease before reaching the collector [27]. Approximately 2 μm decrease was observed in diameters of aligned compared to random sPCL, whereas 0.3 μm in pPCL fibers caused by fiber stretching on the rotating collector [28]. The difference in the patch thickness is caused by the higher average fiber diameter of smooth compared to porous PCL fibers, as the electrospinning time was kept constant for all samples.

Our mechanical testing of PCL membranes revealed similar results to other studies [29,30]. The higher tensile strength of aligned fibers is attributed to polymer chains alignment and stretching during electrospinning and decreased inter-fiber porosity of fiber mats [31,32,33]. The significantly higher mechanical properties of aligned compared to random sPCL fibers confirmed that fiber mats are mechanically anisotropic [34]. In random fibers, the stretching mechanism begins with fibers alignment parallel to the stretching axis, next the fibers are stretched to the max strength while the fiber diameter decreases, leading to fiber necking and final failure, see Movie S1 in the Supplementary Material. The decreased strength of porous compared to smooth fibers is also affected by the lower average fiber diameter that decreases the mechanical properties of PCL fibers [35] and high surface porosity [36], so the tested cross-sectional area is significantly lower which is not included in the calculation of the stress [37]. It demonstrates that the mechanical performance of electrospun fibers depends not only on fiber orientation but also on the interactions and adhesion forces between them [38,39] related to the changes in fibers morphologies or surface properties [40,41]. It was reported that the formation of the mechanical interlocking system strongly depends on surface properties and the presence of crevices, pores, roughness, and irregularities [42].

Mechanical testing of pPCL random fibers in situ in SEM revealed that fibers formed the interlocking systems. While stretching, fibers sliding over their surfaces are locked by the pores that impede their lateral motion and prevent the shear stress [43,44]. Additionally, the locking increases their strain, which was observed with the necking of the fibers leading to their failure (Figure 4A,B). The increased stretching resulted also from the stretching of the individual fibrils within the porous fibers system (Figure 4C,D). Previously, a similar interlocking system was used in designing composites of porous epoxy microparticles (PEM) and epoxidized natural rubber (ENR) and natural rubber (NR) [45]. The interlocking mechanism enhanced the M100 and M300 moduli, and elongation at break that induced an increase in the stiffness of composite of PEM and ENR/NR.

To examine the spreading and controlled release in time we used hemp oil due to its rich contents of EFAs and proteins that are crucial in AD treatment [46]. The spreading of oil was measured on PCL patches deposited onto the gelatin-based skin model, as it shows similar architecture and moisture to human skin [47,48,49]. The greatest spreading area was observed for sPCL-aligned fibers due to its low interfiber porosity (see Figure 2B) that inhibited its penetration deeper into the patch, and oil spreading was limited to the fibers top surface layer (Figure 5D–F). The increased spreading area on pPCL compared to sPCL random fibers was due to the change in wetting properties of smooth and porous PCL fibers [9]. Patches with reduced oil spreading areas showed higher oil release in time, as the oil penetrated inside the 3D patch system. The sPCL aligned patches showed the lowest oil release, due to its high spreading area on the topmost layer of the patch, which limited further oil penetration. Hemp oil showed a great impact on skin moisturization increasing it up to 25% in the case of random pPCL fibers. Results confirmed the beneficial role of hemp oil, as PCL control patches without oil incorporation showed similar results before and after the experiment. This was attributed to the high porosity of PCL membranes [7] that enables free thermoregulation of skin. Volunteer 2, with the driest skin (usually observed in AD) before the experiment shown the best skin moisturization results after the test (Figure 6B). Skin moisturization is critical for successful AD treatment, preventing inflammatory reactions.

The designed patches consisting of the layered sPCL/pPCL fibers increased the hemp oil release on volunteers’ skin up to 55% within 6 h. In previous studies, the patches based on electrospun PVB fibers showed approx. 13% release of evening primrose oil within 1 h [24]. The prolonged-release carriers are in high demand in AD treatment, as the moisturization time is elongated. In electrospun PCL patches, the controlled release for long-term therapies was obtained because of their high surface-area-to-volume ratio, together with good permeability and adequate mechanical properties. Additionally, by patch application, we can prevent external infections and scratching. After 6 h of patch application, the volunteers’ skin showed an increase in moisture up to 20% confirming the beneficial role of hemp oil. Skin moisturizing serves a key role in supporting the regeneration of the damaged skin barrier, lessening the transepiderrmal water loss, maintaining skin hydration, and alleviating the dry skin [2]. Skin moisture is critical in the treatment of AD, reducing the itchiness of the inflamed skin [50]. It was reported that consistent use of moisturizers for dermal hydration abate associating xerosis and pruritus to finally reduce the inflammation and the necessity of topical steroid applications [51,52].

## 5. Conclusions

In this study we produce smooth and porous PCL fibers deposited randomly and aligned. The mechanical testing of membranes showed significantly higher tensile strength of aligned compared to random sPCL fibers because of polymer chain alignment and stretch during electrospinning and decreased inter-fiber porosity of fiber mats. Interestingly, similar tensile strength was observed for random and aligned pPCL, as the mechanical testing of pPCL fibers in situ in SEM showed that porous fibers form the interlocking system then increase their tensile properties. We designed the PCL patches consisting of aligned sPCL and random pPCL fibers, showing up to 55% oil release within 6 h. The measurement of skin moisture on human skin showed that the great impact of hemp oil on skin moisturization increased up to 20%. Our results confirmed that electrospun PCL membranes are great natural oil carriers with adequate mechanical properties that can provide the easy to apply patches for skin. The designed systems allow for long-term, controlled oil release, which is crucial in AD treatment, providing a solution that can be developed further for funding the best strategies in medical care.

## Figures and Tables

**Table 1:** Electrospinning parameters for smooth (sPCL) and porous (pPCL) fibers.

Sample	Fiber Orientation	Voltage [kV]	Flow Rate [mL·h^−1^]	Distance between Nozzle and Collector [cm]	T [°C]	RH [%]	Collector Rotation Speed [rpm]
sPCL	random	14	0.5	15	25	40	-
sPCL	aligned	14	0.5	15	25	40	1500
pPCL	random	14	1	20	25	70	-
pPCL	aligned	14	1	20	25	70	2500

**Table 2:** The average values from mechanical testing of the PCL fiber mats showing: σ_max_ tensile strength, Ɛ_max_ strain at max strength, and Ɛ_f_ strain at failure.

Sample	Fiber Orientation	*σ*_max_ [MPa]	*Ɛ*_max_ [%]	*Ɛ*_f_ [%]
**sPCL**	random	0.51 ± 0.1	98.36 ± 19.0	524.96 ± 154.4
	aligned	0.78 ± 0.2	490.28 ± 346.1	913.69 ± 109.4
**pPCL**	random	0.16 ± 0.003	154.48 ± 50.5	395.46 ± 48.5
	aligned	0.16 ± 0.001	83.50 ± 35.0	238.14 ± 89.3

## Supplementary Materials

The following are available online at https://www.mdpi.com/2077-0375/11/1/26/s1. Figure S1: Cross-sectional SEM images of PCL samples after freeze-fracture: (A,B) sPCL and (C,D) pPCL random and aligned patches respectively. Figure S2: Mechanical testing module with tensile tested fibers. Figure S3: (A) Gelatin-based skin model cast in Petri-dish, utilized for oil spreading tests and (B) ESEM image of skin model topography. Figure S4: PCL patches applied on the skin of volunteers’ forehand. Movie S1: showing mechanical testing of PCL fibers under stereo microscope. Movie S2: showing the interaction between connected pPCL fibers during the tensile testing.
