# Supplementary material for: Electrospun PCL Patches with Controlled Fiber Morphology and Mechanical Performance for Skin Moisturization via Long-Term Release of Hemp Oil for Atopic Dermatitis
Source: Membranes (Basel). 2020 Dec 31;11(1):26. doi: 10.3390/membranes11010026 (PMC7824198; doi:10.3390/membranes11010026)
Supplement: Supplementary file 1 [file membranes-11-00026-s001.zip › membranes-1031930-SI.pdf]

membranes

Supporrting Information:

Electrospun PCL Patches with Controlled Fiber Morphology and Mechanical Performance for Skin Moisturization via Long-Term Release of Hemp Oil for Atopic Dermatitis

Sara Metwally, Daniel P. Ura, Zuzanna J. Krysiak, Łukasz Kaniuk, Piotr K. Szewczyk and Urszula Stachewicz *

Faculty of Metals Engineering and Industrial Computer Science, AGH University of Science and Technology, 30-059 Kraków, Poland; metwally@agh.edu.pl (S.M.); urad@agh.edu.pl (D.P.U.); krysiak@agh.edu.pl (Z.J.K.); kaniuk@agh.edu.pl (Ł.K.); pszew@agh.edu.pl (P.K.S.)

* Correspondence: ustachew@agh.edu.pl

Keywords: PCL; electrospinning; fibers; tensile strength; hemp oil; skin patches; release; skin moisture; atopic dermatitis

The Supplementary Material include the following information:
- Figure S1. showing SEM images of PCL samples cross-section after freeze-fracture,
- Figure S2. showing the mechanical testing module,
- Figure S3. showing the gelatin-based skin model and ESEM image of the cast film,
- Figure S4. PCL patches applied on volunteers' forehand skin,
- Movie S1. showing mechanical testing of PCL fibers under a stereo microscope,
- Movie S2. showing the interaction between connected pPCL fibers during the tensile testing.

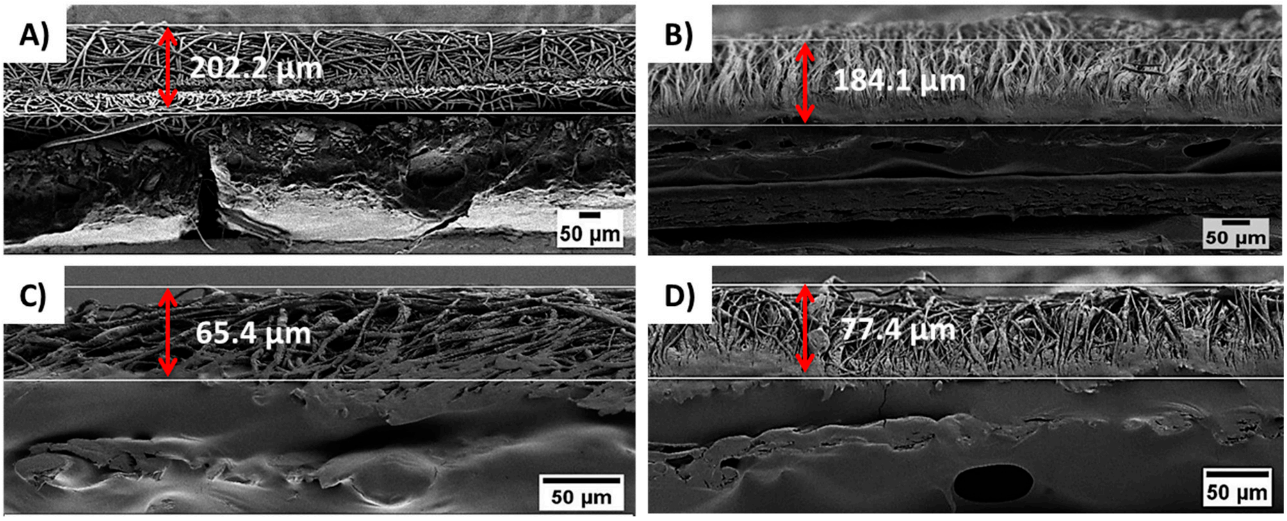

Figure S1. Cross-sectional SEM images of PCL samples after freeze-fracture: (**A**–**B**) sPCL and (**C**–**D**) pPCL random and aligned patches respectively.

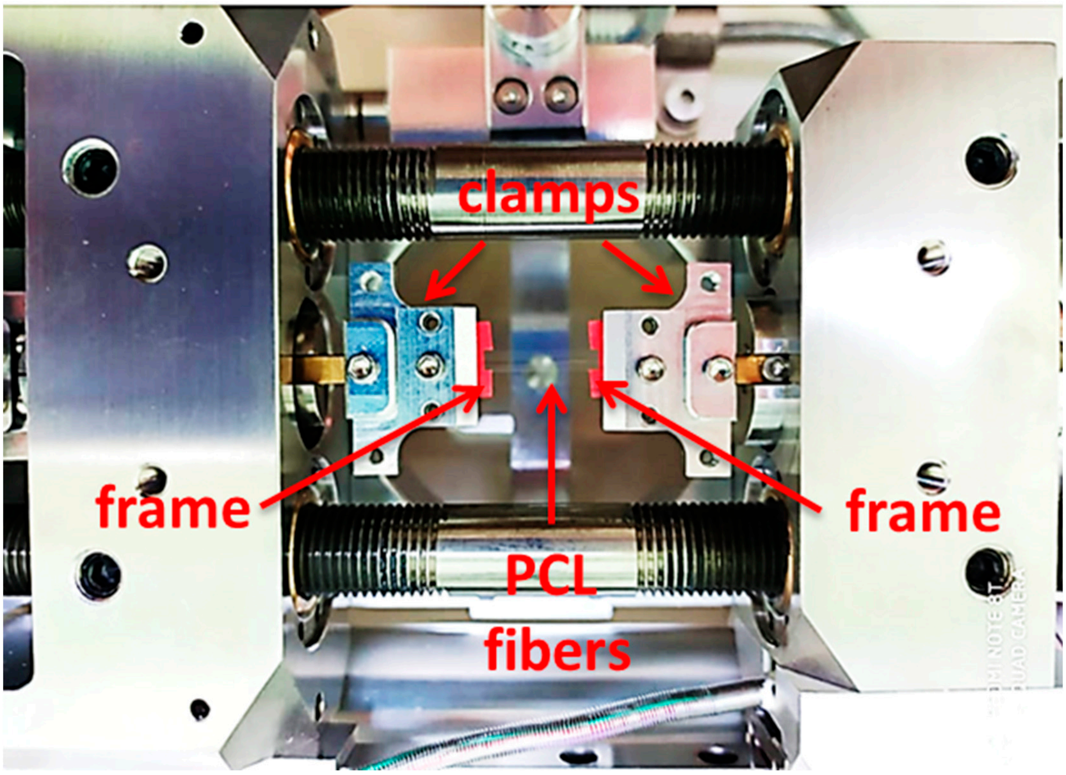

Figure S2. Mechanical testing module with tensile tested fibers.

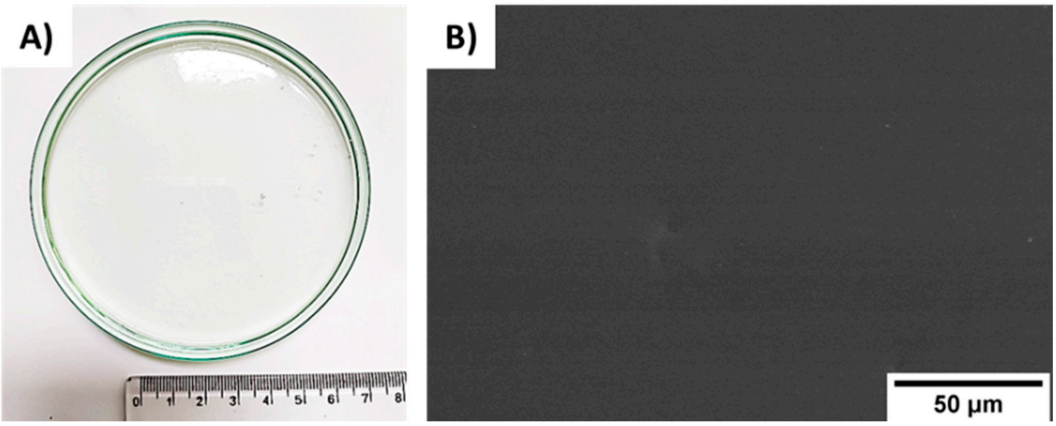

Figure S3. (**A**) Gelatin-based skin model cast in Petri-dish, utilized for oil spreading tests and (**B**) ESEM image of skin model topography.

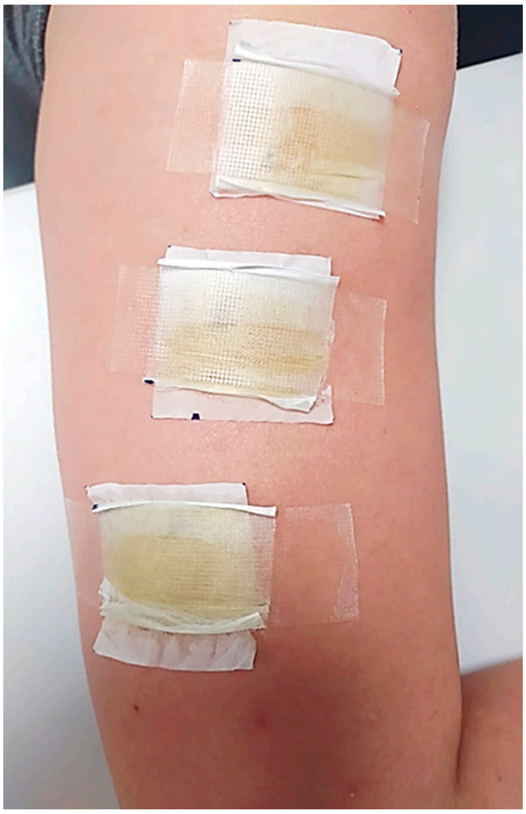

Figure S4. PCL patches applied on the skin of volunteers' forehand.
