# Supplementary figures and images for: Electrospun PCL Patches with Controlled Fiber Morphology and Mechanical Performance for Skin Moisturization via Long-Term Release of Hemp Oil for Atopic Dermatitis
Source: Membranes (Basel). 2020 Dec 31;11(1):26. doi: 10.3390/membranes11010026 (PMC7824198; doi:10.3390/membranes11010026)

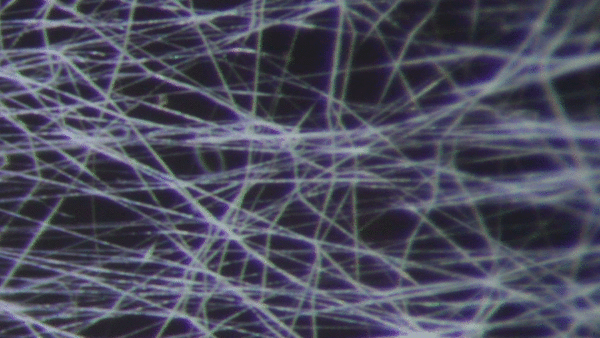

Supplement: Supplementary file 1 [file membranes-11-00026-s001.zip › Movie S1.gif]

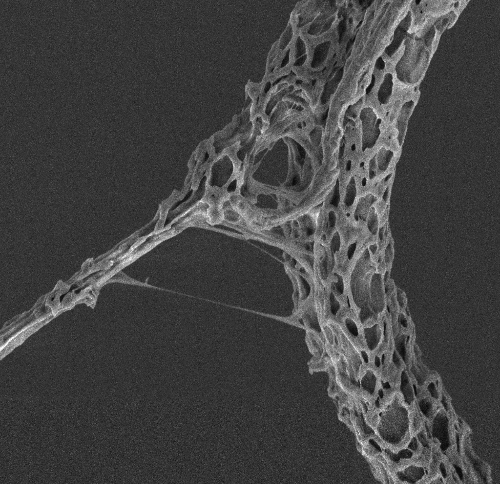

Supplement: Supplementary file 1 [file membranes-11-00026-s001.zip › Movie S2.gif]
